# Investigational Research: Timeline, Trials, and Future Directions of Spinal Disc Arthroplasty

**DOI:** 10.7759/cureus.16739

**Published:** 2021-07-29

**Authors:** Brian Fiani, Jacob M Nanney, Akash Villait, Manraj Sekhon, Thao Doan

**Affiliations:** 1 Neurosurgery, Desert Regional Medical Center, Palm Springs, USA; 2 College of Medicine, University of Kentucky, Lexington, USA; 3 Osteopathic Medicine, Midwestern University Arizona College of Osteopathic Medicine, Glendale, USA; 4 Medicine, Oakland University William Beaumont School of Medicine, Rochester, USA; 5 School of Medicine, University of Texas Medical Branch, Galveston, USA

**Keywords:** disc arthroplasty, artificial disc, degenerative spine disease, lumbar disc degeneration, cervical disc degeneration, interbody device, range of motion, pseudoarthrosis, adjacent segment disease, hybrid

## Abstract

Spinal disc arthroplasty (SDA) has been a rising alternative to fusion for the treatment of degenerative disc disease (DDD). This review aims to provide an overview of the timeline, approvals, and limitations of SDA through analysis of U.S. Food and Drug Administration (FDA)-approved trials. Clinical studies have shown more successful outcomes when comparing cervical disc arthroplasty (CDA) with anterior cervical decompression and fusion, with the key benefits of decreased risk of nerve root compression and adjacent disc disease. CDA is currently approved by the FDA for one- and two-level disc pathologies. However, there are no approved trials for three-level or more cervical pathologies. The FDA approved its usage for the treatment of one-level lumbar disc pathologies in 2007 and recently approved two-level disc pathologies in 2020. Thoracic SDA has not been approved by the FDA, and there are no currently occurring clinical trials. While multilevel SDA has been approved in the cervical and lumbar spine, it has not been approved in more than two adjacent vertebral levels. Based on these clinical studies, future research is needed to compare the success of SDA for three-level or more disc pathologies. There have been recent publications showing promising results, though no FDA-approved clinical trials exist. Furthermore, a hybrid construct has been a recent surgical method to treat multilevel DDD. In this approach, arthroplasty and fusion techniques are combined in alternating fashion at adjacent levels to treat two- and three-level disc disease. Hybrid arthroplasty compared with SDA is currently being studied in clinical trials. As such, long-term research with FDA-approved clinical trials is needed to understand the benefits and limitations of different approaches in the treatment of DDD.

## Introduction and background

Anterior cervical discectomy and fusion (ACDF) has historically been the gold standard for treating degenerative disc disease (DDD) in the cervical spine. However, more recently, cervical disc arthroplasty (CDA) has become more prominent as an alternative to ACDF for treating cervical DDD due to its ability to preserve segmental motion and decrease the risk of adjacent disc disease along with nerve root compression [1]. Long-term clinical studies on single-level treatments have even demonstrated superior clinical outcomes for CDA when compared with ACDF [2].

Currently, CDA is only approved by the U.S. Food and Drug Administration (FDA) for the treatment of one- and two-level cervical pathology with established long-term safety and effectiveness [1]. Naturally, the question of whether CDA can be used for the treatment of three levels in the cervical spine arises. Similarly, spinal disc arthroplasty (SDA) has been approved for usage in one- and two-level lumbar disc pathology with encouraging results. These approvals beg a similar question: If and when will there be trials and approvals for a three-level disc arthroplasty? In the interim, hybrid constructs have been rising in popularity with the combination of arthroplasty and fusion techniques in alternating adjacent levels. Herein, we set out to discuss the use of disc arthroplasty and the timeline of its approval processes, which includes cervical and lumbar spine DDD. Treating multilevel DDD has been associated with complications, such as adjacent-level disease and pseudoarthrosis, when using the traditional fusion methods. We press forth the question of the three-level arthroplasty. The encouraging results of SDA surgeries to date should inspire the ambition to break new boundaries with courageous enthusiasm for future possibilities.

## Review

Timeline

SDA was first approved in the lumbar spine by the FDA on October 26, 2004 (Table 1) [3]. The first artificial disc to receive FDA approval in the United States was the Charite artificial disc by DePuy Spine, Inc. (Raynham, USA) [3]. This device was approved for single-level use at L4-S1 in patients with DDD. The Charite artificial disc was shown to be safe and effective for the treatment of DDD (Table 2) [4]. The Charite was also shown to have equivalent or better outcomes compared to alternative controls [4]. On July 16, 2007, the FDA approved SDA in the cervical spine [3]. The first artificial disc to receive FDA approval for cervical disc arthroplasty in the United States was the Prestige cervical disc system by Medtronic Sofamor Danek, Inc. (Memphis, USA) [3]. This device was approved for single-level use at C3-C7 for intractable radiculopathy and/or myelopathy [3]. The Prestige cervical disc system has been shown to be effective in reconstructing the cervical spine and in preserving normal motion [5].

**Table 1:** FDA approval of cervical and lumbar artificial discs.

Approval date	Device	Cervical or lumbar	Level
October 26, 2004	Charite	Lumbar	One, L4-S1
August 14, 2006	ProDisc-L	Lumbar	One, L3-S1
July 16, 2007	Prestige	Cervical	One, C3-C7
December 17, 2007	Prodisc TM-C	Cervical	One, C3-C7
May 12, 2009	Bryan	Cervical	One, C3-C7
October 26, 2012	Nuvasive PCM	Cervical	One, C3-C7
August 7, 2013	Mobi-C	Cervical	One, C3-C7
August 23, 2013	Mobi-C	Cervical	Two, C3-C7
July 24, 2014	Prestige LP	Cervical	One, C3-C7
June 11, 2015	activL	Lumbar	One, L4-S1
July 7, 2016	Prestige LP	Cervical	One or two, C3-C7
February 6, 2019	M6-C	Cervical	One, C3-C7
April 10, 2020	Prodisc-L	Lumbar	One or two, L3-S1
September 18, 2020	Simplify	Cervical	One, C3-C7
April 1, 2020	Simplify	Cervical	One or two, C3-C7

**Table 2:** Key steps in FDA approval of spinal disc arthroplasty.

Date	Key step
October 26, 2004	First spinal disc arthroplasty approved by the FDA (Charite for lumbar spine)
July 16, 2007	First cervical disc arthroplasty approved by the FDA (Prestige for cervical spine)
August 23, 2013	First multilevel spinal disc arthroplasty approved by the FDA (Mobi-C for two contiguous levels in the cervical spine)
April 10, 2020	First multilevel lumbar disc arthroplasty approved by the FDA (Prodisc-L for one or two contiguous levels in the lumbar spine)

On August 23, 2013, the FDA approved CDA with Mobi-C (Zimmer Biomet Spine, Inc, Westminster, USA) in the United States for two contiguous levels at C3-C7 [3]. The Mobi-C was approved for use in two contiguous levels in patients with intractable radiculopathy or myelopathy [3]. The Mobi-C became the first artificial disc approved by the FDA for use at multiple levels [3]. A comparative study found no significant difference between one- and two-level arthroplasty with Mobi-C with regards to clinical and radiological outcomes [6]. On April 10, 2020, the FDA approved lumbar disc arthroplasty (LDA) with Prodisc-L (DePuy Synthes, West Chester, USA) total disc replacement for one or two adjacent vertebral levels from L3-S1 [3]. The Prodisc-L total disc replacement was approved for use in patients with DDD [3]. A comparative study found no significant difference between one- and two-level arthroplasty with Prodisc-L [7]. SDA has not yet been approved for more than two adjacent vertebral levels. Initial clinical trials involving three or more contiguous vertebral levels are currently underway and literature largely supports this movement. Thoracic SDA is not yet approved by the FDA. To the best of our knowledge, no clinical trials involving thoracic SDA are underway.

Future directions

Although prospective randomized trials have compared one- and two-level CDA with an approach such as the ACDF, there have yet to be similar trials comparing the treatment of three or more level disc disease or spondylosis. In a prospective, a consecutive series of 229 prosthetic implantations was concurrently compared as single vs. multilevel cervical arthroplasty (53 double, 12 triple, and 4 quadruple levels). They found mean improvement of neck disability index (NDI) for single cases (37.6%) vs. multilevel cases (52.6%) (*p *= 0.021), visual analogue scale (VAS), and Odom’s scores, while the reoperation rates were similar between the two groups [8]. One retrospective study followed 50 patients who received three-level CDA at C3-C7 compared to an age- and sex-matched group of 50 patients who underwent ACDF at C3-C7. For the follow-up period of 24 months, clinical outcomes such as neck and arm pain VAS scores, modified Japanese Orthopaedic Association (mJOA) scores, and NDI scores were similar between both groups, while the CDA series had increased range of motion [9]. Another retrospective study examined 139 cervical disc arthroplasties (116 three-level and 23 four-level). From preoperative period to seven years postoperatively, patient-reported outcomes improved (*p *< 0.001), including mean NDI (by 45.9%), the Veterans Rand 12-item Health Survey (by 54.1%), and mental component summary (by 41.85%), with an 88% patient satisfaction rate [1]. Furthermore, Buttacavoli et al. retrospectively reviewed 43 patients treated for three-level disc degenerative disease, comparing those who were given artificial disc (ProDisc-L) or circumferential fusion. On average, there were three fewer hospital days for patients treated with artificial disc replacement (4.77 ± 1.11 days) vs. fusion (8.00 ± 1.82 days) (*p* < 0.0001). This study’s cost analysis showed that artificial disc replacement was 54% less than fusion, including instrumentation costs [10].

There has been increased interest in the use of multilevel LDA. However, clinical trials and studies have mainly examined one- and two-level disc replacement, but few have focused on three or more multilevel LDAs. Seeking to evaluate clinical and radiological outcomes, Rasouli et al. prospectively followed 159 patients with two-level (n = 114), three-level (n = 41), or four-level (n = 4) total lumbar disc replacement at 6 weeks, 3 months, 6 months, and 24-72 months postoperatively. There were no mean differences in the range of motion between the groups except for L5-S1. All groups had a reduction in Oswestry disability index (ODI) and VAS scores (*p *< 0.05). Additionally, a relatively more recent approach to treating multilevel DDD is to perform hybrid arthroplasties alongside fusions, which involve either implanting the discs adjacent to fusion at the same time during surgery or adjacent to a prior fusion. Only three repairs were needed within 72 months [11]. Furthermore, a relatively more recent approach to treating multilevel DDD is to perform hybrid arthroplasties alongside fusions, which involve either implanting the discs adjacent to fusion at the same time during surgery or adjacent to a prior fusion. In a retrospective analysis, Chen et al. reviewed 64 patients with chronic low back pain who were grouped into multilevel hybrid constructs (two- or three-level vs. anterior lumbar interbody fusion (ALIF) alone). The hybrid construct group showed improved VAS and ODI scores (52.2%/50% vs. 28.3%/25.5%). Kaplan-Meier analysis demonstrated 80.5% survivorship for hybrids vs. 75.9% for ALIF, five years postoperatively [12]. Similarly, Scott-Young et al. prospectively followed 617 patients undergoing hybrid surgery for chronic back pain and found a clinically significant reduction in back and leg pain throughout the months followed for up to eight years. The mean ODI and VAS score difference is above minimally meaningful clinically important difference = 10, suggesting meaningfulness [13].

Appropriately blinded, randomized clinical trials are needed to demonstrate the safety and efficacy of three or more levels of arthroplasties in the spine. For example, a new, ongoing, prospective, single-blind, randomized controlled trial is comparing CDA vs. ACDF of one or multilevel cervical DDD on the basis of costs, effectiveness, and utility. This trial will examine adjacent segmental disease, reoperation rate, non-inferiority parameters, NDI, VAS for neck and arm pain, Hospital Anxiety Depression Scale, generic quality of life, and the mJOA score at baseline and then every six months for four years [14]. Additionally, performing multilevel arthroplasties in the thoracic spine is developing, so further observational studies are desired to observe its radiological and clinical outcomes. Therefore, there is a need for longer-term data and higher-level trial evidence to support utilizing hybrid constructs for three or more multilevel disc arthroplasty interventions for multilevel disc pathologies.

## Conclusions

SDA was first approved by the FDA in 2004 and continues to be a viable alternative to standard decompression and fusion methods for the treatment of DDD. Clinical studies have shown that CDA preserves segmental motion while decreasing the risk of adjacent disc disease and nerve root compression when compared to ACDF. Likewise, lumbar arthroplasties have been shown to be safe and effective. The FDA has approved treatment of one- and two-level cervical and lumbar disc pathologies, but there are no approved trials for thoracic SDA. We speculate this is due to the low incidence of disc disease in the thoracic spine, but it could be a foreseeable future treatment method. New studies have already shown excellent outcomes with three-level or more disc arthroplasties at contiguous levels, and the next step in the process is the FDA approval. New surgical methods, such as hybrid construct, are combining decompression and fusion with arthroplasty to treat multilevel DDD with comparable results to disc replacement alone. However, more data are needed to further assess hybrid construct compared to SDA for multilevel disc pathologies. Future studies, with the use of long-term randomized controlled trials or observational studies with larger patient sample sizes, are needed to evaluate the efficacy of SDA vs. more traditional fusion methods.
